# A panel of hepatitis C virus glycoproteins for the characterization of antibody responses using antibodies with diverse recognition and neutralization patterns

**DOI:** 10.1016/j.virusres.2024.199308

**Published:** 2024-01-11

**Authors:** Ana Chumbe, Marloes Grobben, Joan Capella-Pujol, Sylvie M. Koekkoek, Ian Zon, Stefan Slamanig, Sabrina J. Merat, Tim Beaumont, Kwinten Sliepen, Janke Schinkel, Marit J. van Gils

**Affiliations:** a Amsterdam UMC, University of Amsterdam, Department of Medical Microbiology and Infection Prevention, Amsterdam, the Netherlands; b Amsterdam Institute for Infection and Immunity, Infectious Diseases, Amsterdam, the Netherlands; c AIMM Therapeutics, Amsterdam, the Netherlands

**Keywords:** HCV, HCV pseudoparticle, Virus panel, E1E2 glycoprotein, Monoclonal antibody, Neutralization, Binding, Competition

## Abstract

A vaccine against Hepatitis C virus (HCV) is urgently needed to limit the spread of HCV. The large antigenic diversity of the HCV glycoprotein E1E2 makes it difficult to design a vaccine but also to fully understand the antibody response after infection or vaccination.

Here we designed a panel of HCV pseudoparticles (HCVpps) that cover a wide range of genetically and antigenically diverse E1E2s. We validate our panel using neutralization and a binding antibody multiplex assay (BAMA). The panel of HCVpps includes E1E2 glycoproteins from acute and chronically infected cases in the Netherlands, as well as E1E2 glycoproteins from previously reported HCVs.

Using eight monoclonal antibodies targeting multiple antigenic regions on E1E2, we could categorize four groups of neutralization sensitive viruses with viruses showing neutralization titers over a 100-fold range. One HCVpp (AMS0230) was extremely neutralization resistant and only neutralized by AR4-targeting antibodies. In addition, using binding antibody multiplex competition assay, we delineated mAb epitopes and their interactions. The binding and neutralization sensitivity of the HCVpps were confirmed using patient sera. At the end, eleven HCVpps with unique antibody binding and neutralization profiles were selected as the final panel for standardized HCV antibody assessments.

In conclusion, this HCVpp panel can be used to evaluate antibody binding and neutralization breadth and potency as well as delineate the epitopes targeted in sera from patients or candidate vaccine trials. The HCVpp panel in combination with the established antibody competition assay present highly valuable tools for HCV vaccine development and evaluation.

## Highlights

• Description of a HCVpp panel to evaluate antibody binding and neutralization capacity.
• The HCVpp panel shows a wide range of neutralization diversity including AMS023, an extremely neutralization resistant HCVpp.
• Antibody binding in a bead-based binding antibody multiplex assay (BAMA) using membrane bound E1E2 glycoproteins positively correlate with HCVpp neutralization capacity.
• Binding antibody multiplex competition assay (BAMCA) delineates monoclonal antibody epitopes and their interactions on the E1E2 glycoprotein.

## 1 Introduction

Globally, 58 million people are infected with Hepatitis C virus (HCV) and about 1.5 million new infections occur each year (World Health Organization, n.d.). Despite the availability of direct acting antiviral (DAA) treatment with high success rates, 290,000 HCV-associated deaths occur each year worldwide, as treatment does not reach a large number of patients in need (WHO guidelines, 2018). In addition, DAA treatment does not prevent new infections (Lambers et al., 2011; Simmons et al., 2015) nor cure patients with advanced liver fibrosis or cirrhosis (Ioannou et al., 2019; Kanwal et al., 2017). Thus, there is an urgent need to develop a vaccine that prevents new infections with the ultimate goal to eliminate HCV by the year 2030, a global goal set by the WHO in 2016 (World Health Organization, 2023). Vaccine candidates include protein subunit (Vietheer et al., 2017), virus-like particle (VLP) (Christiansen et al., 2019), nanoparticle vaccines (Capella-Pujol et al., 2022; Sliepen et al., 2022), viral vector (Swadling et al., 2014), peptide (Dawood et al., 2019) and DNA vaccines (Masavuli et al., 2019), however, only two vaccines have entered the clinical trial testing phase and none have shown sufficient efficacy so far (Editorial, 2021; Hartlage and Kapoor, 2021; Page et al., 2021).

For most viral vaccines, the main correlate of protection is the presence of antibodies with sufficient breadth to stop heterologous infections (Plotkin, 2010). Two main features of antibodies are relevant for protection: quantity and functionality (Plotkin, 2010). For example, in humanized liver mice, a high concentration of antibodies has been associated with protection against an HCV challenge (Law et al., 2008) and could even clear an established infection (Pestka et al., 2007). In humans, a rapid induction of neutralizing antibodies (Pestka et al., 2007) and increased neutralization breadth (Kinchen et al., 2018) are associated with spontaneous HCV clearance. These antibodies target multiple antigenic regions on the E1E2 glycoprotein. One of the key targets on E1E2 is the CD81 binding site, as CD81 serves as one of the primary entry receptors for HCV. CD81 interfering antibodies primarily include antibodies that target antigenic region 3 (AR3). AR3, targeted by monoclonal antibodies (mAbs) AR3B (Law et al., 2008) and AT1209 (Merat et al., 2016), overlaps with the CD81 binding domain. Other AR3-targeting mAbs partially overlap with domain D, which is represented by the mAb HC84.26 or partially recognize residues in domain B as well as domain C/AR2 (such as mAb AT1211 (Merat et al., 2016)). Antigenic region 4 (AR4) is targeted by many of the most potent antibodies against HCV, including AR4A^49^ and AT1618 (Merat et al., 2019), and recent reports indicate that they target E2 exclusively when the E1E2 complex is correctly folded^50^. Antigenic site 412 (AS412) (Potter et al., 2012) includes a linear epitope between residues 412 and 423, which is targeted by antibodies with a broad reactivity (such as AP33 (Potter et al., 2012)). The E1 stem also includes an antigenic site between residues 313 and 327 which is targeted by mAb IGH505 (Torrents de la Peña et al., 2022). Other conformational epitopes such as antigenic domain A do not elicit neutralizing antibodies such as mAb CBH4B (Z.-Y. Keck et al., 2004).

Besides neutralization, antibodies have a wide variety of functions through their Fc-domain such as antibody dependent cellular phagocytosis (ADCP), antibody dependent cellular cytotoxicity (ADCC) and complement dependent cytotoxicity (CDC). Antibody effector functions have been recognized to play a key role in antiviral vaccine immunity (Chung et al., 2015) and protection (Gunn et al., 2019) for other viruses, for example against Influenza virus (Sedova et al., 2019). In HCV-infected patients, a strong correlation between ADCC and higher levels of anti-E2 IgG in plasma has been found (Adhikari et al., 2021); however, the role of antibody effector functions in the pathogenesis of HCV is still unknown. As HCV vaccine candidates progress, standardized in vitro assays to study and compare immune responses after natural infection and vaccination, using a representative panel of the antigenic diversity of HCV glycoprotein E1E2 to evaluate immunological responses, are increasingly relevant.

Antibody functionality has been mostly studied by determining their capacity to neutralize viruses using replicating cell culture viruses (HCVccs) (Bankwitz et al., 2020) or pseudoparticles (HCVpps) (Osburn et al., 2014; Urbanowicz et al., 2015). HCVccs are chimeric replicating viruses which often need adaptive mutations in order to be infectious in cell culture (Mathiesen et al., 2015). Constructing novel variants in this system is not a simple task. In contrast, HCVpps are formed by incorporating full-length HCV E1E2 onto lenti- or retroviral core particles, which makes them more flexible to study new sequences or mutations. Despite many differences between the two systems, e.g. lower 50% inhibitory concentration (IC_50_) obtained in the HCVpp system, there is a remarkable positive correlation between the two systems when comparing different antibodies or viral variants (Wasilewski et al., 2016), which is very similar to HIV-1 neutralization assays where the pseudovirus assay is now the standard (Montefiori et al., 2018; Seaman et al., 2010). Other antibody functions including effector functions such as ADCP, ADCC and CDC can also be studied using in vitro cell based assays (Forthal, 2018; Von Holle and Anthony Moody, 2019). However, these have not been widely used for HCV and therefore are not standardized resulting in high inter-assay variability (Brown et al., 2017). A high throughput biophysical platform, such as the Luminex bead-based multiplex immunoassay, could be a good alternative, as this platform provides an economical, quick and robust method to detect binding of antibodies to different antigens as well antigen-specific FcγR binding to quantify antibody effector functions in polyclonal serum responses. Similar assays have been used for immune surveillance of *Streptococcus pneumonia* (Danka et al., 2018), Influenza virus (Germeraad et al., 2019), SARS-CoV-2 (Grobben et al., 2021; Zhu et al., 2022), Human papilloma virus (Opalka et al., 2003) and other pathogens (Danka et al., 2018; Varela et al., 2018; Yufenyuy et al., 2022).

HCV is extremely genetically diverse and has been divided into several genotypes (Simmonds et al., 2005). For HCV, similar to HIV (Dan H. Barouch, 2008) or Influenza virus (Pica and Palese, 2013), heterologous protection is believed to be key to counteract the vast diversity of the virus (Bailey et al., 2019). Therefore, the availability of viruses and E1E2 glycoprotein panels representing global HCV diversity for the different antibody assays are essential. A few HCV E1E2 panels including genetically and antigenically diverse pseudoviruses have been designed to evaluate neutralizing antibody responses (Salas et al., 2022; Urbanowicz et al., 2015). However, due to the huge diversity of HCV, continuous evolution and the lack of relevant clinical information associated with available E1E2 sequences, many of these different panels are not a good antigenic representation or consist of a large set of viruses. In addition, for binding assays such as the bead-based multiplex immunoassay, recombinant glycoproteins, virus lysates or purified membrane bound glycoproteins are needed. Technical difficulties in HCV glycoprotein production cause a significant limitation on the availability of representative E1E2 glycoproteins.

Therefore, well-characterized panels of antigenically diverse E1E2 glycoproteins should be developed for both HCVpps and binding assays. Here, we present a panel of HCV E1E2s that can be used to evaluate the quantity and functionality of antibodies (neutralization and effector functions) in sera after infection or vaccination. Twenty different HCV E1E2s were characterized, using sequences from acutely as well as chronically infected patients, infected by different transmission routes, using monoclonal antibodies targeting all major antigenic regions with diverse potencies. From these 20 HCVpps, a panel of 12 was selected to be further characterized in a multiplexed binding assay to delineate interactions between antigenic regions and validate their compatibility in the different assays. The final panel consists of 11 HCV E1E2s, representing the antigenic and genetic global diversity of HCV, and can be used for the evaluation of HCV antibody breadth and functionality.

## 2 Material an methods

### 2.1 HCVpps selection

A set of 20 E1E2 sequences from six different genotypes were included, nine were from Amsterdam, the Netherlands. Five of the Amsterdam isolates were previously obtained from different clusters of viruses circulating among HIV-infected men who have sex with men (MSM) with HCV infection: AMS0229, AMS0230, AMS0231, AMS0232 and AMS0233 (Genbank ID: OL855834.1, OL855838.1, OL855836.1, OL855837.1 and OL855835.1, respectively); the other four were previously isolated from chronically infected non-MSM individuals: AMS.1b.k2, AMS.2b.k21, AMS.3a.k26, AMS.4dk9 (Genbank ID: KR094962.1 KR094963.1, KR094964.1 and ON623878, respectively). The participants provide informed consent and the study was approved by the Academic Medical Center Institutional Medical Ethics Committee. The remaining E1E2 sequences came from the Nottingham panel (*n* = 10) and H77 as a reference strain (AAB67037 including three amino acid changes at the following positions: R564C, V566A, and G650E). We made a selection of HCVpps based on their neutralization sensitivity: UKNP1.2.3, UKNP2.1.1, UKNP2.2.1, UKNP2.4.1, UKNP3.2.1, UKNP3.2.2, UKNP4.1.1, UKNP5.2.1, UKNP6.1.1, UKNP6.1.2 (Genbank ID: KU285154.1, KU285209.1, KU285211.1, KU285213.1, KU285218.1, KU285219.1, KU285220.1, KU285226.1, KU285227.1 and KU285228.1, respectively). In addition, JFH1 as HCVcc and HCVpp derived from clone JFH1-AM120 (KF700370.1) were included as controls.

### 2.2 HCVpp production

HCVpps were produced as previously described (Chumbe et al., 2022). Briefly, Human embryonic kidney 293T (HEK-293T) cells, maintained in Dulbecco's modified essential medium (DMEM, Gibco by Thermo Fisher Scientific) supplemented with 10% fetal bovine serum (FBS), 1% nonessential amino acids (NEAA) and 0.1% Penicillin-Streptomycin, were transfected using 6 μg of total DNA, 12 μl of Lipofectamine 2000 and Opti-MEM (Invitrogen by Thermo Fisher Scientific) per 10 cm dish. We used the Murine Leukemia Virus (MLV) Gag-Pol, a plasmid encoding firefly luciferase and plasmids encoding E1E2 to produce the HCVpps (Bartosch et al., 2003). These three plasmids were used in optimized ratios as reported previously (Chumbe et al., 2022). As a negative control, we generated pseudoparticles transfected in the absence of E1E2 plasmid. Twenty-four hours after transfection, the media was replaced with DMEM supplemented with 10% fetal bovine serum and 0.1% Penicillin-Streptomycin DMEM. Media containing the HCVpps was harvested 48 h later, passed through a 0.45 µm filter and stored at 4 ⁰C for direct use or −80 ⁰C for storage.

### 2.3 Production of mAbs, soluble CD81 LEL and CD81-Fc proteins

Monoclonal antibodies (mAbs) targeting the main antigenic regions (ARs) were included in this study. AR4A and AT1618 (AR4), AR3B and AT1209 (AR3), HC84.26 (domain D, that share some residues with domain B), AP33, IgH505, AT1211 and CBH4B (Giang et al., 2012). Production of mAbs was done in-house in HEK-293F cells, as described previously (Chumbe et al., 2022). Briefly, heavy and light chain plasmids (1:1 ratio) in a 1:3 ratio with 1 mg/L PEImax (Polysciences) were transfected into HEK-293F cells at a density of 1 million cells/mL in FreeStyle medium (Gibco). The recombinant IgG antibodies were isolated from the cell supernatant after five daysusing a protein A/G column (Thermo Fisher Scientific). First, the cell suspension was centrifuged 25 min at 4000 rpm, and the supernatant was filtered using 0.22 µm pore size SteriTop filters (Millipore). The filtered supernatant was run over a 10 mL protein A/G column followed by two column volumes of PBS wash. The mAbs were eluted from the column using 9 ml of 0.1 M glycine pH 2.5 (elution buffer) in 1 ml of 2 M Tris, pH 8.6 (neutralization buffer). Buffer exchange in PBS and concentration was performed using Vivaspin 100 kDa filters (Sartorius). Antibodies were aliquoted and stored at −20 °C for long-term storage or kept at 4 °C for short-term storage.

The soluble version of the long external loop of the CD81 (CD81 LEL) receptor was used to characterize the availability of the CD81 binding site on the HCVpps. Soluble CD81 LEL with Strep-tag plasmid was kindly provided by Dr. Joe Grove (Grove et al., 2008). It was produced and purified following the same procedure as the mAbs in HEK-293F cells. Total DNA transfected was 312.5 µg per 1 × 10^9^ cells/L. After transfection, protein was harvest from the supernatant six days later and purified by Strep-tactin. CD81 LEL was mixed with 1 M Tris/HCl, pH 8.0 1.5 M NaCl, 10 mM EDTA (1:10 ratio). After supernatant filtration, 2 ml of Biolock per 500 ml 293F supernatant was added and incubated for >15 min. In parallel, a StrepTactinXT column was washed 3 times with 3 ml of 1 M Tris/HCl, pH 8.0 1.5 M NaCl, 10 mM EDTA. Once the column was ready, the supernatant was left to slowly (3 s between drops) run over the column at 4 ᵒC. After 3 times washing with 1 M Tris/HCl, pH 8.0 1.5 M NaCl, 10 mM EDTA, the column was eluted with 1 M Tris/HCl, pH 8.0 1.5 M NaCl, 10 mM EDTA, 50 mM biotin. Eluted protein was later concentrated on PBS with Vivaspin 10 kDa filters (Sartorius).

The CD81-Fc, CD81 LEL (F113-K201) fused to the C terminal of human Fc receptor of IgG1 (E99-K330) using a spacer (GGGGSGGGGS), was cloned in the expression vector AbVec2.0-IGHG1 (Addgene, plasmid #80,795). It was produced in HEK-293F cells and purified using protein A/G column as described above for monoclonal antibody purification.

### 2.4 Biotinylation of mAbs and soluble CD81-Fc protein

AR4A, AT1618, AR3B, AT1209, HC84.26, AP33, IGH505,AT1211, CBH-4B mAbs and CD81-Fc protein were biotinylated using EZ-Link™ Sulfo-NHS-LC-Biotin (Thermo Scientific) following manufacturer instructions. Briefly, 5.5 mg/ml of biotin was diluted and mixed in a 1:4 ratio with 1 mg/ml mAbs or CD81 protein and left overnight at 4 ᵒC. Purification and buffer exchange to PBS was performed the next day using Vivaspin 10 kDa filters (Sartorius). Biotinylated mAbs or protein were stored at 4ᵒC until further use.

### 2.5 HCVpp neutralization assay

Antibody neutralization capacity was determined following the protocol optimized previously in our laboratory (Chumbe et al., 2022). Briefly, 1.5 × 10^4^ Huh-7 cells were seeded in DMEM (Gibco by Thermo Fisher Scientific) supplemented with 10% FBS, 1% NEAA and 0.1% Penicillin/Streptomycin and left over night at 37 °C in 5% CO_2_. HCVpps and mAbs were mixed and kept for one hour at 37 °C in 5% CO_2_ before incubation (30 μl) on top of the seeded Huh-7 cells. After four hours, 200 μl of DMEM 10% FBS, 1% NEAA, 0.1% Penicillin/Streptomycin was added for cell maintenance. Readout was done 72 h after media removal and a 5-minute shaking incubation at room temperature according to the manufacturer's instructions (Luciferase Assay System, E1500 Promega). GloMax Luminometer from Promega was set to dispense 50 µl of luciferase reagent and subsequently the relative light units (RLUs) were directly recorded for one second (integration time) after zero seconds delay.

The ability of soluble CD81LEL to bind and neutralize HCVpps was evaluated similar to mAb neutralization protocol. Briefly, 100 μg/ml of CD81LEL in eight dilution steps (1:3) were added to HCVpps and incubated for one hour at 37 ᵒC in a final volume of 30 μl.

### 2.6 Membrane bound E1E2 production and purification

HEK-293T^CD81KO^ cells were transfected using 40 μg of E1E2 expression plasmids, 80 μl of Lipofectamine 2000 and Opti-MEM (Invitrogen by Thermo Fisher Scientific) per T150 flask. Three days after transfection, the supernatants were removed and the cells were washed twice with PBS before detaching them with Trypsin-EDTA. Non-transfected HEK-293T^CD81KO^ cells were taken along as a negative control. Cell suspensions were spun down and washed with PBS before counting them. Cells were resuspended in 1% Triton buffer (50 mM tris pH 8.0 + 150 mM NaCl + 1% Trition) with 1x proteinase inhibitor cocktail (Thermo Scientific), 1 ml per 1 × 10^6^ cells. After 30 min incubation at 4 ᵒC with rotation, cell lysates were centrifuged at 4000 g at 4 ᵒC for 30 min.

Membrane bound E1E2 (mbE1E2) purification was done with Galanthus Nivalis Lectin (GNL) (Vector laboratories, l-1240–5) columns. Briefly, cell lysates of transfected and non-transfected cells were diluted 1:3 with PBS and added to previously washed GNL columns after which the columns were washed three times with PBS + tritonX100 0.1%. mbE1E2 was eluted from the column with 1.0 M alpha-d-manno-pyranoside in PBS pH 7.5. The mbE1E2 were concentrated using Vivaspin 100 KDa filters (Sartorius).

### 2.7 mbE1E2 coupling to Luminex beads

Each purified mbE1E2 was covalently coupled to one Magplex bead region (Luminex Corporation) using a two-step carbodiimide reaction. Briefly, 40 μl of each mbE1E2 was coupled to 2.5 × 10^6^ Magplex beads (Luminex). Magplex beads were washed with 100 mM monobasic sodium phosphate pH 6.2 and activated by addition of Sulfo-N-Hydroxysulfosuccinimide (Thermo Fisher Scientific) and 1-Ethyl-3- (3-dimethylaminopropyl) carbodiimide (Thermo Fisher Scientific) for 30 min on a rotor at room temperature (RT) in the dark. Activated beads were washed three times with 50 mM MES (Thermo Fisher Scientific) pH 5.0 before addition of mbE1E2 diluted in 245 μl of 50 mM MES pH 5.0. The mix containing the beads and mbE1E2 was incubated for 3 h on a rotor at room temperature in the dark before washing with PBS to elute any unbound protein. Subsequently, the beads were incubated with blocking buffer (PBS containing 2% BSA, 3% FBS, 0.02% Tween-20) for 30 min on a rotator at RT. Beads were then washed and stored with 0.05% Sodium Azide in PBS at pH 7.0.

Prefusion stabilized trimeric RSV-fusion glycoprotein (McLellan, 2013; Grobben et al., 2022) and a native-like secreted form of H77 E1E2 heterodimer (Guest et al., 2021) were used as positive controls; and GNL purified non-transfected lysate (NTL) was used as negative control. 7 μg of each protein and 40 μl of NTL were coupled to 2.5 × 10^6^ Magplex beads.

### 2.8 Luminex assay

The binding of antibodies to the mbE1E2 proteins coupled to the Magplex beads was studied as described previously (Keuning et al., 2021). Briefly, in blocking buffer (PBS containing 2% BSA, 3% FBS, 0.02% Tween-20), 750 coupled beads per region were incubated with 1 μg/ml of mAb in a total volume of 100 μl at 4 ᵒC with rotation overnight in the dark. Subsequently, plates were washed twice with TBS containing 0.05% Tween-20 (TBST) using a hand-held magnetic separator. Beads were then resuspended in 50μl of blocking buffer with Streptavidin-PE (Invitrogen) at a final concentration of 1.3 ng/ml. After 2 h incubation at RT with rotation in the dark, beads were washed twice with TBST using a hand-held magnetic separator. Finally, the beads were resuspended in 70 μl Bioplex sheath fluid (Bio-Rad) and after few minutes in rotation at RT, readouts were performed on the Bioplex 200 (Bio-Rad).

For the cross-competition Luminex assay, we included as competitors IgH505, AP33, AR3B, AT1209, HC84.26, AT1211, AR4A, AT1618, CBH4B (Z.-Y. Keck et al., 2004), CD81-Fc and blocking buffer only. Briefly, in 50 μl of blocking buffer, 750 coupled beads per region were incubated with 25 μl (10 μg/ml) competitor mAb for one hour in the dark. Subsequently, 25 μl of biotinylated mAbs (analytes) at a final concentration of 1 μg/ml (and four dilution steps, 1:10) were added and left at 4 ᵒC with rotation in the dark overnight. Plates were read out as described above. Resulting median fluorescence intensity (MFI) values were corrected by subtraction of MFI values from buffer and beads-only wells. MFI obtained with beads coupled with non-transfected purified products were also subtracted before proceeding with the analysis.

We used the binding at 1 μg/ml to calculate residual binding, because at this concentration RSV-F and NTL beads still showed very low binding and most mAbs gave high MFI values. A value above 10x the highest median value (8.5 MFI) of the NTL beads to the biotinylated mAbs was used as a threshold for binding. Residual binding was determined considering the non-competitor as 100%. Reproducibility of the results was confirmed by performing replicate runs.

### 2.9 Heatmap clustering

To identify antigenic similarity between different HCVpps within our panel, a heatmap was created using the “heatmap.2″ tool of the gplots package of the statistical environment R (“Heatmap hierarchical Clustering,” n.d.) (http://www.hiv.lanl.gov/content/sequence/HEATMAP/heatmap.html) largely used for sera and monoclonal antibodies (Binley et al., 2004). Two hierarchical clustering algorithms were used to group HCVpps (rows) based on neutralization (IC_50_s) and binding results (MFI). To assess the stability of the clusters we used bootstrapping with 1000 iterations. For neutralization, logarithmic values in base 10 of the IC_50_s were used. The threshold values 0.023 μg/ml and 50 μg/ml were used for the IC_50_s. For binding, MFI values for each E1E2-mAb combination were used after subtraction of the background (NTL-mAbs). Graphs were displayed using GraphPad Prism version 8.3.0.

### 2.10 Evolutionary relationships

A phylogenetic tree of the full E1E2 amino acid sequences was inferred using the Neighbor-Joining method (Saitou and Nei, 1987). Evolutionary distances were computed using the Poisson correction method (Troadec et al., 1998) and using the number of amino acid substitutions per site as unit. The percentage of replicate trees in which the associated taxa clustered together in the bootstrap test (1000 replicates) was calculated (Felsenstein, 1985). All ambiguous positions were removed for each sequence pair. Phylogenetic analysis was conducted in MEGA6 (Tamura et al., 2013).

### 2.11 Principal component analysis

Log transformed IC_50_s were used to perform principal component analysis (PCA) analysis with Graphpad Prism 9.1.0. PCA was based on a correlation matrix and the percentage of total explained variance was used to select the principal components (PCs).

### 2.12 Statistical analyses

IC_50_s were calculated using log (inhibitor) vs response (variable slope) considering 0% and 100% as bottom and top constraints. Spearman's rank correlation matrices were generated for all HCVpps tested using IC_50_s of eight mAbs and for all mAbs plus CD81 LEL using IC_50_s of 20 HCVpps. Neutralization (IC_50_s) and binding (MFI) geometric means (GMT) were calculated and used for linear regressions using Pearson r. GraphPad Prism version 9.1.0. was used for all statistical analysis.

## 3 Results

### 3.1 Characterization of a HCVpp panel with a wide range of antibody neutralization sensitivities

**Fig. 1:**
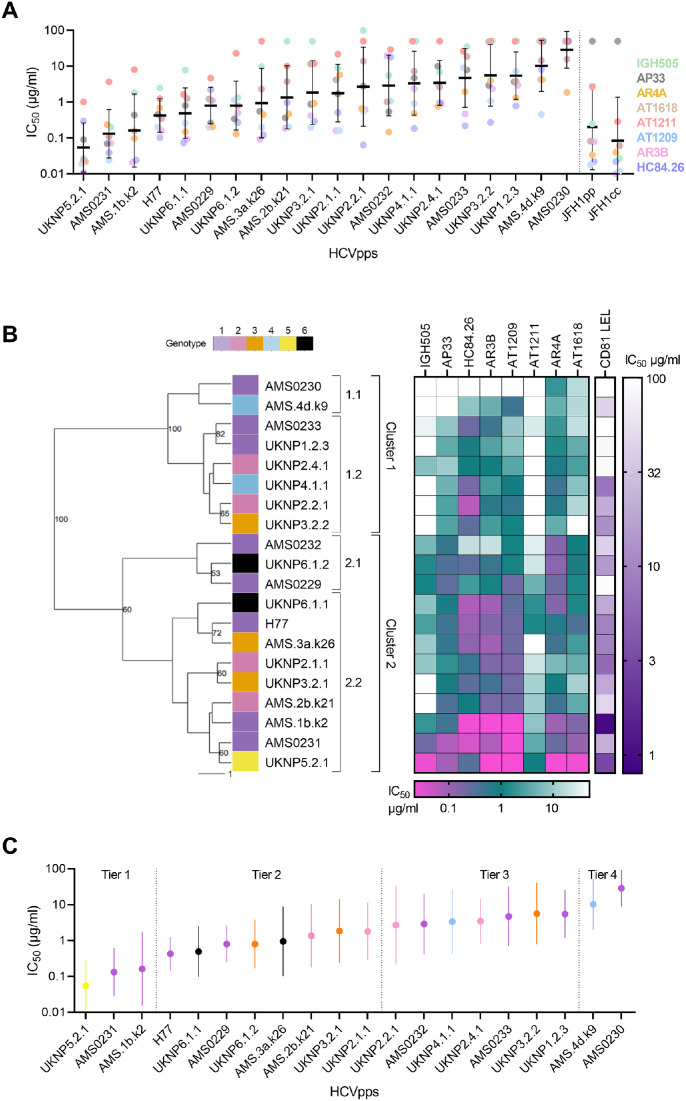
HCV pseudoparticle panel. (A) Differences in HCVpp sensitivity to eight mAbs: AR4A, AT1618, IGH505, AP33, AT1211, AR3B, AT1209 and HC84.26 (color coded per mAbs in figure). HCVpps are ordered low to high based on their geometric mean neutralization titer (GMT). GMT and standard deviation per HCVpp are depicted as a black line and error bar. A vertical dotted line separates our controls JFH1pp and JFH1cc from our HCVpp panel. Each mAb-HCVpp combination was tested two or more times on different days. (B) A dendogram of 20 HCVpps (color coded per genotype 1 to 6) based on their sensitivity to neutralization against eight mAbs is shown. The IC_50_ values (μg/ml) per mAbs and CD81 LEL per HCVpp are shown with horizontal and vertical color scale bars for mAbs and CD81, respectively. HCVpps were grouped into two main clusters (1 and 2) using hierarchical clustering based on the individual neutralization IC_50_ values. Bootstrap resampling (1000 iterations) was applied, nodes with support above 50% are shown. (C) Tier classification of HCVpp panel color coded per genotype 1 to 6 (see legend in panel B). Dots indicate mean IC_50_ values and the whiskers indicate standard error of the mean per HCVpp. HCVpps are arranged from most sensitive to most resistant based on the mean IC_50_. HCVpps are divided into four groups (Tier 1 - 4) which are indicated by dotted lines.

We used eight monoclonal neutralizing antibodies (mAbs) - targeting antigenic region (AR) 3 (AR3B, AT1209), AR4 (AR4A, AT1618), domain D (HC84.26), domain C/AR2 (AT1211), Antigenic site 412 (AP33) on E2 and the AR on E1 (IGH505) - to antigenically characterize 20 HCVpps. These HCVpps represent six major genotypes, selected from previously published HCVpps and E1E2 sequences and from local HCV infected individuals without prior knowledge on their neutralization sensitivity. We also included JFH1 E1E2 as pseudoparticles (JFH1pp) and cell culture (JFH1cc) and showed that the neutralization sensitivity between these two was highly correlated, with the HCVpp showing overall more resistance (Supplementary Fig. 1). The geometric mean titer (GMT) of the 50% inhibitory concentration (IC_50_) of all eight mAbs for each HCVpp showed a wide range of neutralization sensitivities between the different HCVpps (Fig. 1A). Some HCVpps were highly susceptible to neutralization, i.e. UKNP5.2.1 and AMS0231, with a GMT of 0.055 μg/ml and 0.13 μg/ml respectively, whereas others were extremely resistant, such as AMS0230, with a GMT of 28.7 μg/ml (Fig. 1A). One of the most sensitive (AMS0231) and also the most resistant to neutralization (AMS0230) were among the nine novel HCVpps derived from local circulating HCV variants. A wide range in sensitivity to the individual mAbs was observed, indicating clear differences in antigenicity within the HCVpp panel.

IC_50_s of mAbs targeting the same ARs were highly correlated (Supplementary Fig. 2A). For example, AR4-targeting mAbs (AT1618 and AR4A) and AR3-targetting mAbs (AR3B and AT1209) indeed showed string correlations (Spearman *r* ≥ 0.83). However, no clear separation between mAbs targeting different ARs was observed (Supplementary Fig. 2A). For example, for AR4-targeting mAbs, especially AT1618, a high correlation was also found with AT1209, AR3B, AT1211 and IGH505 (Spearman *r* = 0.67, *r* = 0.7, *r* = 0.76, *r* = 0.9, respectively and *p*<0.001 for all cases). Interestingly, IgH505, a mAb targeting a linear epitope at the stem of E1, correlated not only with AR4-targeting mAbs (AT1618 and AR4A, Spearman *r* ≥ 0.73 *p*<0.001 for all cases), but also with AT1209, AR3B, AT1211 (Spearman *r* = 0.57 [*p* = 0.005], *r* = 0.64 [*p* = 0.001] and *r* = 0.76 [*p*<0.001], respectively). AP33 showed no correlation with any other mAb. Using hierarchical clustering based on the monoclonal IC_50_ values, HC84.26, AT1209 and AR3B clustered together but AR4A and AT1618 did not (Supplementary Fig. 2B). This indicates that HCVpps are overall more sensitive or resistant to antibody neutralization irrespective of the target epitope.

In addition, we measured the neutralization activity of CD81 LEL (recombinant protein of the large-extracellular loop of the HCV receptor CD81) against all 20 HCVpps. CD81 LEL did not neutralize AMS0230 and surprisingly, did not neutralize AMS0233, a HCVpp sensitive to neutralization by AR3-targeting mAbs, which overlap the CD81 binding domain (Fig. 1B). CD81 LEL only neutralized AMS0229, UKNP1.2.3 and UKNP2.4.1 HCVpps at very high concentrations (IC_50_ of 92 μg/ml, 85 μg/ml and 82 μg/ml, respectively), even though these HCVpps are sensitive to AR3-targeting mAbs (Supplementary Fig. 3). Overall, CD81 LEL IC_50_ values positively correlated with the GMT of all the eight mAbs (Spearman *r* = 0.670) (Supplementary Fig. 4A) with higher correlation values for the individual antibodies HC84.26, AR3B and AT1209 that target AR3 that overlap with the CD81 binding site (Supplementary Fig. 4B–D). CD81 LEL IC_50_s also correlated with AR4A and AT1618, AT1211 and IGH505 IC50 values (Supplementary Fig. 4E–H), but weaker, but did correlate with AP33 (Supplementary Fig. 4I).

To further delineate our HCVpps panel, they were grouped into two main clusters (1 and 2) using hierarchical clustering based on the individual neutralization IC_50_ values (Fig. 1B). Cluster 1 included more neutralization resistant HCVpps and could be further divided into two subclusters with high confidence (bootstrap 100). Cluster 2 included more neutralization sensitive HCVpps and could also be divided into two subclusters with sufficient statistical support (bootstrap 60). In contrast to the phylogenetic tree of the 20 E1E2 amino acids, which showed clustering by genotype (Supplementary Fig. 5), the antigenic (sub)clusters 1 and 2 contained multiple and mixed genotypes, underscoring that antigenic clustering is independent of genotype.

Besides the hierarchical clustering of the HCVpps as described above, we determined if the HCVpps could also be ordered by their overall neutralization sensitivities. All HCVpps were ranked according to the mean of the log_10_ IC_50_ for all eight mAbs combined, similar to Salas et al. (2022) (Salas et al., 2022). HCVpps were classified in four groups based on the mean and the standard deviation (SD). Group 1 or tier 1 contains 3 extremely sensitive HCVpps, with mean IC_50_s more than one SD below the mean (IC_50_ <0.31 μg/ml), group/tier 2 contains 8 HCVpps with mean IC_50_ s not more than one SD below the mean, (0.31 μg/ml < mean IC_50_ < 1.48 μg/ml), group/tier 3 HCVpps contains seven HCVpps, with mean IC_50_s not more than one SD above the mean (1.48 μg/ml < mean IC_50_ < 6.90 μg/ml) and finally group/tier 4 contains two extremely difficult to neutralize HCVpps (mean IC_50_s > 6.90 μg/ml) (Fig. 1C). The clustering based on individual neutralization sensitivity (Fig. 1B) was very similar to the tier categorization; with the tier 1 and 2 HCVpps all in cluster 2 and the tier 3 and 4 HCVpps in cluster 1 except AMS0232 which was classified as tier 3 but clustered together with the tier 2 HCVpps in cluster 2, most likely due to the high sensitivity of AMS0232 to AR4A.

### 3.2 Twelve HCVpps distributed in four neutralization tiers form a representative virus panel with high antigenic and genetic diversity

**Fig. 2:**
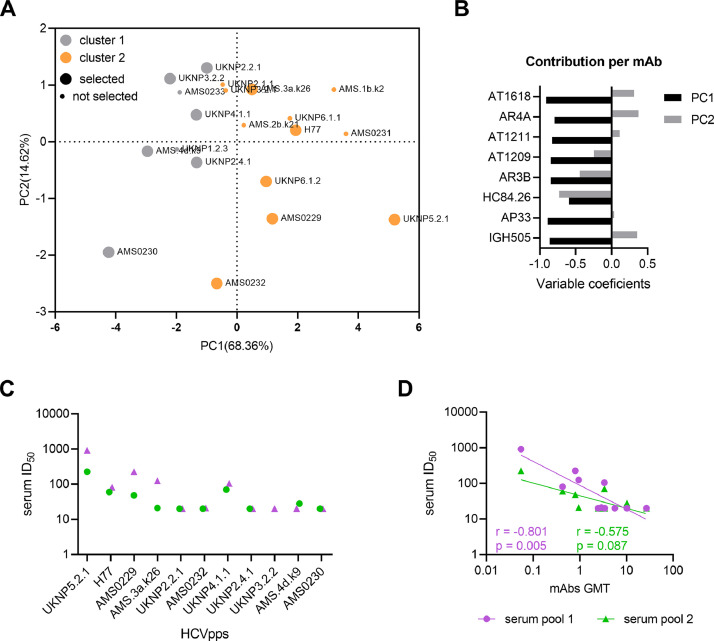
Relationship among HCVpps based on neutralization sensitivity. (A) Principal component analysis (PCA) of HCVpps based on neutralization IC_50_ values. First two principal components (PC1 and PC2) are plotted and percentage of variation captured by each PC is indicated in brackets. HCVpps from cluster 1 and 2 are colored in gray and orange, respectively. HCVpps selected for the final panel are in bigger circles compared to the non-selected HCVpps. (B) Loading bar plots from the PCA loadings of the mAbs for PC1 and PC2. (C) Serum pool ID_50_ values against the selected HCVpps. HCVpps are arranged from most sensitive to most resistant based on the mAb sensitivity (Fig. 1). (D) Correlation between the serum pool ID_50_ values and mAbs geometric titer (GMT) against the different HCVpps are shown.

In order to avoid redundancy in our panel of HCVpps, we constructed a correlation matrix and performed a principal component analysis (PCA) based on the IC_50_ values of the mAbs. We aimed to select the minimal number of HCVpps whilst maintaining antigenic diversity. A correlation matrix enables the identification of HCVpps with unique antibody sensitivity profiling and a PCA helps to summarize in two variables most of the information contained in the larger data set. The correlation matrix in Supplementary Fig. 6 displayed HCVpps with highly similar sensitivity to the panel of mAbs but also several HCVpps with very unique fingerprints within both antigenic clusters (AMS0230, UKNP2.4.1, UKNP5.2.1, AMS0232 and AMS0229 (Supplementary Fig. 6). Similar to the hierarchical clustering, the two clusters were nicely separated by the principal component (PC) 1 which captured nearly 69% of the variation in the dataset (Fig. 2A). All mAbs contributed equally to PC1, while for PC2 the direction was different for AR4A, AT1618 and IGH505, compared to the other mAbs (Fig. 2B).

We made a final selection for the HCVpp panel based on unique neutralization sensitivity and tier clustering, antigenic clustering, and representation of different genotypes using the PCA and correlation matrix. We selected genotype 1a strains H77, AMS0229, AMS0230 and AMS0232, genotype 2 strains UKNP2.2.1 and UKNP2.4.1, genotype 3 strains UKNP3.2.2 and AMS.3a.k26, genotype 4 strains UKNP4.1.1 and AMS.4dk9, genotype 5 strain UKNP5.2.1 and genotype 6 strain UKNP6.1.2. These twelve strains cover a large area of the antigenic distance in the PCA analysis and are represented in all four tier categories. To validate the panel, we tested 2 serum pools: pool 1 (4 sera 3–6 months after primary HCV infection) and pool 2 (4 sera >1 year after primary HCV infection). The two serum pools mostly neutralized viruses that were more sensitive to mAb neutralization (Fig. 2C), which resulted in a significant correlation between serum neutralization and the GMT of the mAbs against the different HCVpps for pool 1 and a near significant correlation due to lower neutralization capacity for pool 2 (Fig. 2D).

### 3.3 Antibody neutralization and binding across HCVpps significantly correlate

**Fig. 3:**
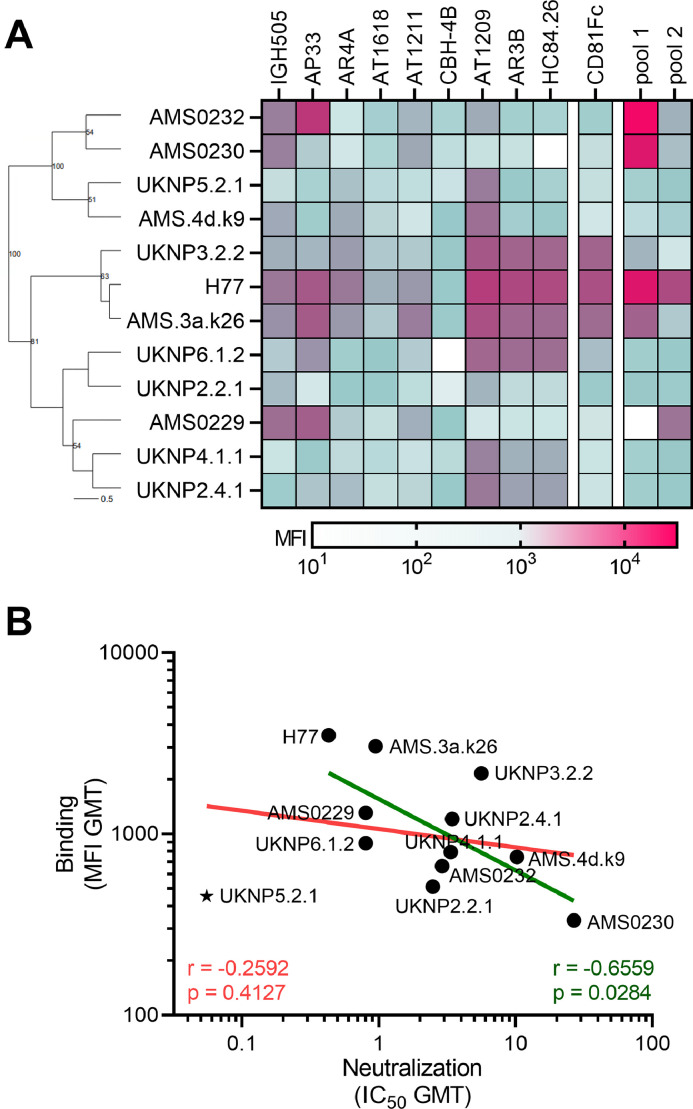
Antibody and serum binding to mbE1E2 in the Luminex assay. (A) A Hierarchical clustering based on the Ward linkage algorithm using Euclidean distance is shown to the left. Nodes with values above 50% are shown. The horizontal line represents the scale for the tree, which reflects the distance or dissimilarity between data points in percentage. On the right, a heat map of median fluoresce intensity (MFI) values per mbE1E2-mAb combination after subtraction of NTL-mAb values is shown as well as for CD81 and the two serum pools. The color scale based on MFI is shown at the bottom with white indicating no binding, green intermediate binding and purple high binding. (B) Linear regression analysis between neutralization (IC_50_ GMT) and binding (MFI GMT) of the 12 HCVpps with (in pink) and without UKNP5.2.1 (in green) are shown. Correlation coefficients (Spearman r) with p values are indicated in the graph including and excluding UKNP5.2.1.

To determine whether the selected twelve HCVpps were also antigenically distinct for antibody binding, we tested the membrane bound E1E2 (mbE1E2) glycoproteins of the selected HCVpps in a Luminex bead-based binding antibody multiplex assay (BAMA) against the eight mAbs tested above as well as CD81 and non-neutralizing antibody CBH4B, whose epitope is exposed on the surface of E2 (Hadlock et al., 2000). Our panel of mAbs showed a dose dependent binding to the HCV mbE1E2 coupled beads but not to the negative control beads loaded with HIV-1 gp120, SARS-CoV-2 spike, RSV F and Influenza virus HA protein (Supplementary Fig. 7). The mAbs showed high binding to neutralization sensitive HCVpps (H77 and AMS.3a.k26) and low binding to neutralization resistant HCVpps (AMS0230 and AMS.4dk9) (Fig. 3A). We observed very similar results for CD81-Fc compared to the AR3-targeting mAbs. The serum pools showed high binding to the genotype 1 mbE1E2, especially the AMS strains, which have been derived from the same cohort as these serum samples. Besides UKNP5.2.1, binding in this assay correlated with the IC_50_ values obtained in the neutralization assays, excluding CBH4B as no neutralization data was obtained for this mAb (Spearman *r*= −0.66, *p* = 0.028 excluding UKNP5.2.1) (Fig. 3B). We excluded UKNP5.2.1 from our HCVpps panel, because of the poor correlation between neutralization and binding, leaving eleven HCVpps in the final panel. These results indicated that antibody binding in general is a good predictor of antibody neutralization using mbE1E2, especially when testing E1E2s from intermediate or high neutralization resistant HCVpps. We have used hierarchical clustering of the mAb binding to E1E2 in the BAMA (Supplementary Fig. 8). Interestingly, the mAbs targeting the same binding domain (HC84.26, AT1209, AR3B and CD81-Fc) did not cluster together but appeared in three different clusters. This suggesting no clear delineation of antigenic regions based on neutralization (Supplementary Fig. 2B) or binding data.

### 3.4 Binding antibody multiplex competition analysis reveal novel interactions of antibody binding to E1E2

**Fig. 4:**
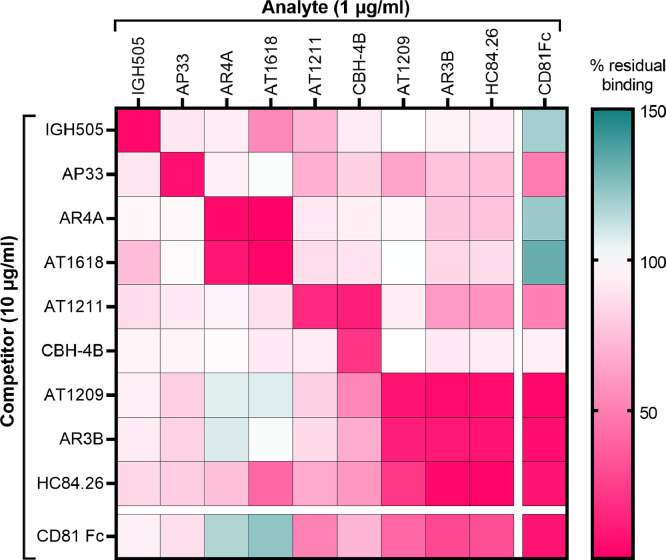
Binding competition between the different monoclonal antibodies. A heatmap of the mean% residual binding for all HCVpps and the eight antibodies as analyte and competitor is depicted (Supplementary Fig. 10 shows the% residual binding per individual HCVpp). The BAMCA data for the different HCVpps and mAbs were from at least two independent experiments which showed very similar% residual binding. Only mbE1E2s-mAb combinations above the background threshold were used to calculate the means. Competitors (vertical) at 10 μg/ml and biotinylated analytes (horizontal) at 1 μg/ml are shown. Autologous competitions are shown in the diagonal. Color coded scale bar is shown on the right, with pink indicating strong competition, white no competition and green, binding enhancement.

Next, we wanted to investigate the effect of antibody binding on the antigenicity of E1E2. We aimed to establish such an antibody competition matrix for the 11 strains used in the HCVpp panel. Therefore, we developed the binding antibody multiplex competition assay (BAMCA), which enabled us to measure mAb competition against multiple different E1E2 proteins in parallel. We included all mAbs used in the neutralization assay, CD81-Fc, as well as CBH4B (a non-neutralizing antibody). After we optimized the BAMCA assay and determined that the threshold could be set at 100 MFI, we excluded 11 competition pairs; 6/11 mbE1E2s for CBH4B, 3/11 mbE1E2s for HC84.26 and 2/11 mbE1E2s for AR3B (Supplementary Fig. 9). We then calculated the mean competition profiles for each individual mbE1E2 (Supplementary Fig. 10) and a single profile for all E1E2-mAbs (Fig. 4) using the percentage of residual binding in the presence of excess competitor mAb. We observed potent self-competition (≤15% residual binding) as highlighted by the low residual binding in the diagonal of all panels.

As expected, mAbs that bind similar epitopes showed strong competition (≤22% residual binding), such as AR4A and AT1618, which target AR4 and HC84.26, AR3B and AT1209, which target AR3/domain D. Similarly, the AR3/domain d-targeting bNAbs strongly competed with CD81. Interestingly, IGH505, which binds a linear epitope on E1, weakly competed with the AR4-targeting mAbs, especially AT1618. Unexpectedly, AR3-targeting HC84.26 also showed competition with AR4-targeting AT1618 and in some cases with AR4A (for UKNP2.2.1 E1E2s). For AR3B, AT1209 and mainly CD81-Fc, we observed the opposite effect, meaning enhanced binding of AR4A and AT1618 when AT1209, AR3B or CD81-Fc were bound. However, the opposite effect (binding enhancement after addition of AR4A or AT1618) was only seen with CD81-Fc. These data indicate that AR3/domain d-targeting mAbs and the CD81 receptor induce allosteric changes that alter the AR4 epitope or the exposure of AR4. Finally, binding of AP33 to E1E2, which targets the base of the hyper variable region 1 (HVR1), showed one-directional competition with reduced binding of most mAbs and CD81, except for IGH505, AR4A and AT1618, and hardly any competition the other way around. AT1211 competed strongly with non-neutralizing CBH-4B Ab against domain A and more weakly, but consistently with domain B/D mAbs AR3B, HC84.26 and CD81-Fc. This is in line with mutational analyses, which indicated that AT1211 targets domain C, which is located next to domain A and close to domain B/D on E2 (Merat et al., 2016; Pierce et al., 2016).

Overall, these results show that this binding antibody multiplex competition assay (BAMCA) is a powerful method to delineate mAb epitopes on HCV glycoproteins from different strains at the same time. Using this BAMCA assay we confirmed that binding of certain mAbs interfere because of epitope proximity, but also discovered novel mAb and epitope interactions. This indicates that BAMCA might be useful to define epitopes of novel mAbs against HCV E1E2 and other viral glycoproteins.

## 4 Discussion

For the detailed evaluation and comparison of antibody responses in HCV infection or vaccination studies, a standard panel of viruses representing genetic and antigenic diversity is needed. Here, we characterized twenty HCVpps, using eight mAbs targeting different antigenic regions with diverse potencies, and selected 11 HCVpps that covered broad genetic and antigenic diversity. The HCVpps showed a large range of neutralization sensitivities, also confirmed with patient sera, from very sensitive to very resistant with positive correlations between antibody neutralization and binding. The HCVpps could be distinguished into two main clusters based on their neutralization sensitivity and further subdivided into four neutralization groups, which were not associated with genotype. Therefore, we propose this final HCVpp panel as a standard panel, including the most prevalent genotypes, to evaluate HCV antibody breadth and functionality.

The binding and neutralization data correlated for all but one E1E2 (UKNP5.2.1). E1E2 is known to be a very fragile, unstable protein (Pfaff-Kilgore et al., 2022) and highly malleable (Toth et al., 2021) and therefore purified mbE1E2s could include a mixture of E1E2 in different conformations (Guest et al., 2021; Torrents de la Peña et al., 2022). The susceptibility to neutralization of UKNP5.2.1 might be due to very low overall protein stability rather than exposure of specific antibody target epitopes. Therefore, our final panel does not include UKNP5.2.1.

Our extended panel includes mostly clinical isolates from the University of Nottingham Trent HCV Cohort study (Urbanowicz et al., 2015) or from sexual and non-sexual transmitted cases isolated in the Netherlands. We did not find a specific genotype associated with neutralization sensitivity or resistance which is in accordance with other studies (Bankwitz et al., 2020; Salas et al., 2022). Our panel of 11 HCVpps includes a balance of most genotypes, from 1, 2, 3, 4 and 6 and some E1E2s overlap between our panel and other available HCVcc (Bankwitz et al., 2020) and HCVpps (Salas et al., 2022; Urbanowicz et al., 2015) panels. For most HCVpps, our neutralization results are in agreement with previous reports (e.g.UKNP2.2.1, UKNP3.2.1, H77 (Salas et al., 2022; Urbanowicz et al., 2015)) except for UKNP2.4.1 and UKNP6.1.1. In our hands using the MLV system, UKNP2.4.1 produced well and was neutralized (IC_50_s <50 μg/ml) by all mAbs except AT1211 and UKNP6.1.1 was neutralized by all mAbs. Others have reported that UKNP2.4.1 and UKNP6.1.1 were not neutralized at 100 μg/ml using similar mAbs but a different HCVpp system (Salas et al., 2022). One explanation could be the difference in HCVpp production yield as it has been observed that differences in pseudoparticle systems influences infectivity (Urbanowicz et al., 2016), which could affect the neutralization sensitivity of these HCVpps. Limiting the number of HCVpps for the subpanel was challenging because several HCVpps showed individual signatures and only small redundancy was observed in our PCA. Nonetheless, with the inclusion of the neutralization resistant AMS230 variant, our final panel of 11 HCVpps has the widest range of neutralization diversity reported so far, and covers the major genotypes using mostly clinical isolates.

AMS0230 is a very interesting clinical isolate as it was highly neutralization resistant. In contrast, the highly similar isolate AMS0231 was sensitive to neutralization. AMS0231 and AMS0230 were isolated from participants infected in 2005–2006 (Thomas et al., 2015). However, AMS0231 virus was isolated at 11 months after the estimated date of infection (and cleared after treatment), while the AMS0230 virus was isolated after a relapse over 4 years after the infection. Similar to AMS0230, AMS.4dk9 was isolated from a chronically infected patient after more than 6 years of infection. It has been observed that viral diversity increases from transition to chronic infection in HCV (Ho et al., 2017) and other related hepaciviruses (Gömer et al., 2022), which is likely the result of escape from the antibody responses by the virus (Merani et al., 2011; Walczak and Mora, 2021). We could speculate that HCV variants isolated later after infection are more neutralization resistant especially if they undergo selective pressure, such as immune response or antiviral treatments. AMS0231 and AMS0230 have 92.6% amino acid similarity in E1E2, suggesting that only a few key amino acid changes likely impact neutralization sensitivity. There are interesting differences in HVR1 as well as in the AR3 and CD81 binding loop, specifically at positions 438, 442, and 528 (Supplementary Fig. 11), which could explain the difference in sensitivity for the AR3-targeting antibodies between these two viruses and the high resistance of AMS0230, however this needs to be further evaluated.

The binding antibody multiplex assay (BAMA) has proven to be highly robust for evaluating antibody responses after coronavirus vaccination (Grobben et al., 2021; van Gils et al., 2022) or natural infections (Sechan et al., 2022) from multiple sources (Keuning et al., 2021). However, this assay has not been tested for HCV before. We designed a robust BAMA as well as BAMCA using mbE1E2s that helped us to study binding as well as competition (BAMCA) between mAbs targeting different antigenic regions. We chose mbE1E2s over soluble E1E2s because soluble proteins that could bind with AR4 mAbs are restricted to a few sequences (Guest et al., 2021). Besides, mbE1E2s include more protein heterogeneity that might better reflect what is naturally present on virions where AR3 and AR4 regions are well presented. A competitive Luminex immunoassay has been used previously to identify different types of Human papilloma virus (Opalka et al., 2003) and it is now recommended by the CDC for serological response studies after HPV vaccination (CDC, 2006). Here, we show that the HCV BAMCA can define mAb epitopes and reveal complex binding features, interactions and relationships within the HCV E1E2. It is highly sensitive so minimal amounts of protein and sera are sufficient to detect binding to multiple E1E2s at once in large screenings before moving forward with cell based assays, such as neutralization assay (Butler et al., 2019).

Several antigenic regions on HCV E1E2 have been identified so far. Although the competition assay provided a clear distinction between antigenic regions, our HCVpps showed an overall positive correlation in neutralization sensitivity independent of the target region. This makes antibody profiling of polyclonal responses to delineate targeted epitopes difficult. A more extensive HCVpp panel in combination with additional mAbs could be considered in addition to our current selection for this type of analysis. HCVpps exhibiting differential sensitivity to mAbs for the different antigenic regions have not been described so far, particularly between AR3 and AR4.

In this study, different degrees of competition between mAbs targeting different antigenic regions were observed, especially for certain E1E2s. We observed binding competition between IGH505 and AT1618. High resolution protein structures revealed that the epitopes for IgH505 and AR4A are in close proximity (Torrents de la Peña et al., 2022), which could cause steric hindrance explaining the competition between IgH505 and AT1618 since AT1618 also targets the AR4 region. The other AR4-targeting mAb AR4A showed less competition with IGH505, which is most likely caused by differences in the angle of approach by the different AR4 mAbs. Because of the likely close vicinity of AT1618 to IGH505, allosteric changes in the IGH505 epitope induced by AT1618 are less likely to explain the competition also since the IGH505 epitope is relatively non-conformational. In addition, competition between AP33 and AR3-targeting mAbs was observed to be asymmetrical, indicating conformational changes after AP33 binding could play a role in the binding of AR3-targeting mAbs as suggested before in HCVcc for other similar mAbs (Z. Keck et al., 2013). CD81-Fc, which strongly competes with domain B/D mAbs, showed bidirectional binding enhancement with AR4-targeting mAbs. Furthermore, the neutralization capacity of AR3-targeting mAbs AT1209 and AR3B correlated and these mAbs competed for binding with domain B/D mAb HC84.26. However, only AR3B and AT1209 (and not HC84.26) lead to a unidirectional binding enhancement of AR4-targeting mAbs for some HCVpps with intermediate sensitivity (tier 2 and tier 3). This might indicate a synergistic effect of binding between both antigenic regions. Interesting, synergy was previously reported between domain B/D and AR4A mAbs by using the HCVcc system (Carlsen et al., 2014). Other studies have found enhanced neutralization breadth when combining mAbs targeting different epitopes (Mankowski et al., 2018; Merat et al., 2016). This may indicate that allosteric interaction between domain B/D mAbs and AR4A mAbs are specifically a product of mAbs that induce conformational changes.

## 5 Conclusion

We presented a panel consisting of 11 HCVpps with a wide antigenic diversity. This neutralization panel has the widest range of neutralization diversity reported so far and was further classified in multiple tiers. The antigenic and genetic diversity and genotype-independent neutralization and binding sensitivity of HCV emphasize the importance of using panels based on antigenic diversity rather than only genotypic diversity to clearly differentiate between vaccine candidates. In addition, the multiplexed mbE1E2 binding and competition assays represent robust methods to evaluate binding of mAbs to decipher target epitopes and predict neutralization potency and breadth, and could be applied for sera as well. By systematically evaluating a set of HCVpps, we have created a panel to study antibody binding and neutralization breadth and potency, especially for the selection of strong neutralization capacity, as well as delineate the different target epitopes, which is highly advantageous for the evaluation of humoral responses in infection or vaccination studies.

## Funding

This work was supported by the Netherlands Organization for Scientific Research (NWO) ZonMw (grant numbers 91719372 and 015.015.042 to J.S.), and by Amsterdam UMC through an AMC fellowship (to M.J.v.G.) and an AMC PhD scholarship (to A.C.). The funders had no role in study design, data collection and analysis, decision to publish, or preparation of the manuscript.

## CRediT authorship contribution statement

**Ana Chumbe:** Writing – original draft, Visualization, Validation, Methodology, Investigation, Funding acquisition, Formal analysis, Data curation, Conceptualization. **Marloes Grobben:** Writing – original draft, Validation, Resources, Methodology. **Joan Capella-Pujol:** Writing – original draft, Resources, Methodology. **Sylvie M. Koekkoek:** Resources, Investigation. **Ian Zon:** Resources, Investigation. **Stefan Slamanig:** Investigation. **Sabrina J. Merat:** Resources. **Tim Beaumont:** Resources. **Kwinten Sliepen:** Writing – review & editing, Visualization, Resources. **Janke Schinkel:** Writing – review & editing, Supervision, Funding acquisition, Conceptualization. **Marit J. van Gils:** Writing – review & editing, Supervision, Funding acquisition, Conceptualization.

## Declaration of competing interest

The authors declare that they have no known competing financial interests or personal relationships that could have appeared to influence the work reported in this paper.

## Data Availability

Data will be made available on request.

## Appendix Supplementary materials

Image, application 1
