# Supplementary material for: A panel of hepatitis C virus glycoproteins for the characterization of antibody responses using antibodies with diverse recognition and neutralization patterns
Source: Virus Res. 2024 Jan 11;341:199308. doi: 10.1016/j.virusres.2024.199308 (PMC10821612; doi:10.1016/j.virusres.2024.199308)
Supplement: Supplementary file 1 [file mmc1.pdf]

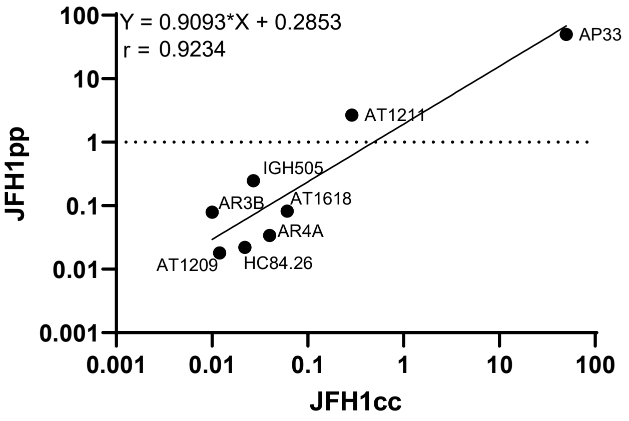

Supplementary Figure 1. Linear regression between JFH1cc (X axis) and JFH1pp (Y axis) IC_{50} values for eight mAbs. The correlation coefficient (Pearson r) between JH1cc and JFH1pp IC_{50} values is shown. IC_{50} values were obtained using two independent experiments.

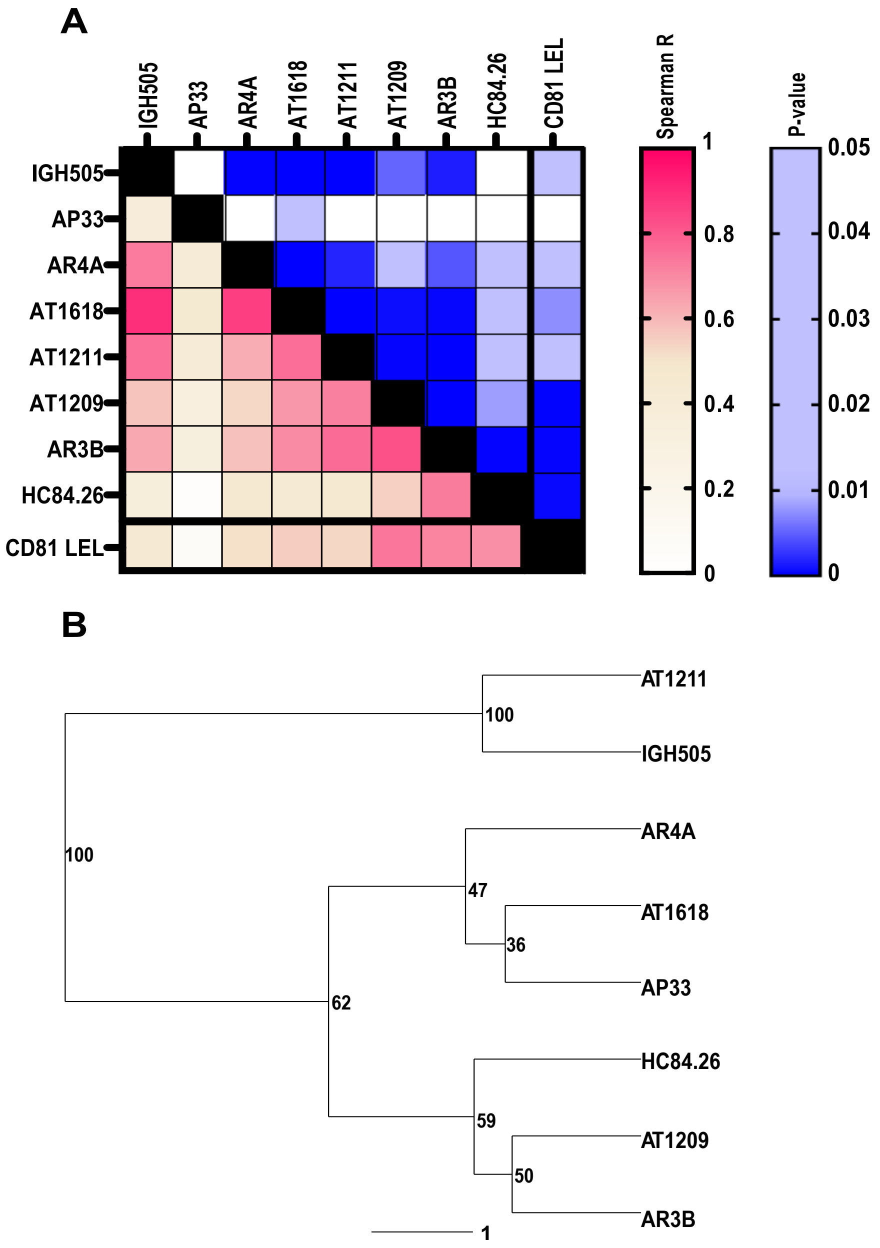

Supplementary Figure 2. Correlation matrix based on neutralization sensitivity (A) Heatmap showing pairwise Spearman's correlation matrix for eight mAbs and CD81LEL [log_{10}GMT IC_{50} values (µg/ml)] using the full panel of 20 HCVpp (minimum of two IC_{50} values per HCVpp-mAb combination). Spearman's correlations and p-values are in red and blue, respectively. (B) Hierarchical clustering of mAbs based on log10 GMT IC_{50} values (µg/ml) is shown. Bootstrap resampling (1000 iterations) was applied, nodes with support above 10% are shown. Horizontal line represents the scale for the tree branches, which reflects the distance or dissimilarity between data points in µg/ml.

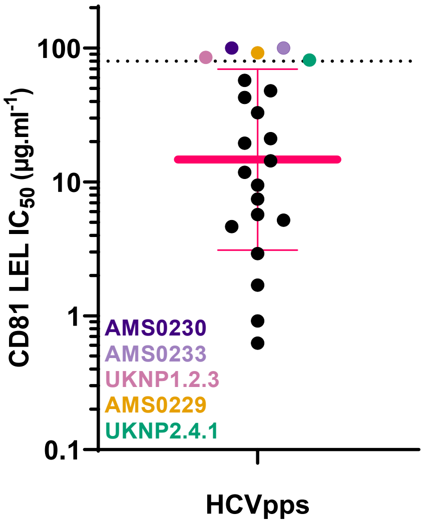

Supplementary Figure 3. HCVpp sensitivity to CD81 LEL. GMT and standard deviation re shown as red lines. HCVpps with IC_{50} values above 80µg/ml (dotted line) or not blocked at the highest concentration tested (100 µg/ml) are highlighted in color.

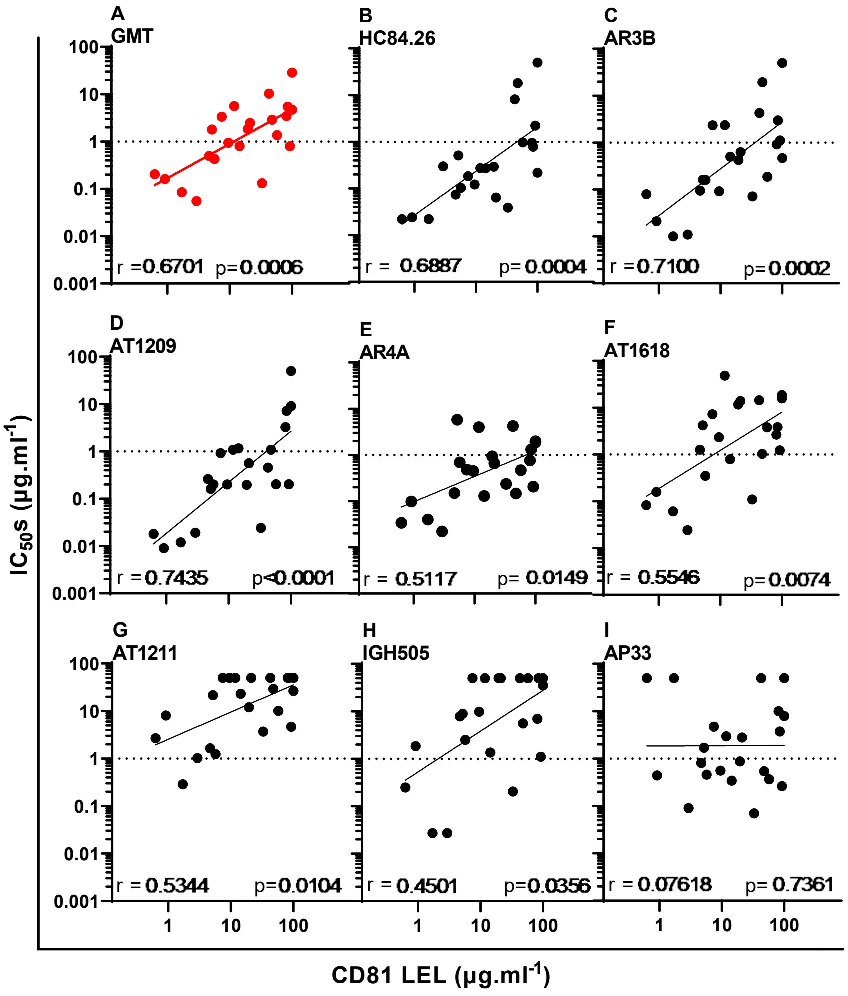

Supplementary Figure 4. CD81 LEL correlations. Spearman r correlations between CD81 LEL (X axis) and GMT of all mAbs (A) in red or individual mAbs (B to I) in black. For A to I, 20 HCVpps are plotted and a horizonal dotted line is depicted at 1 µg/ml.

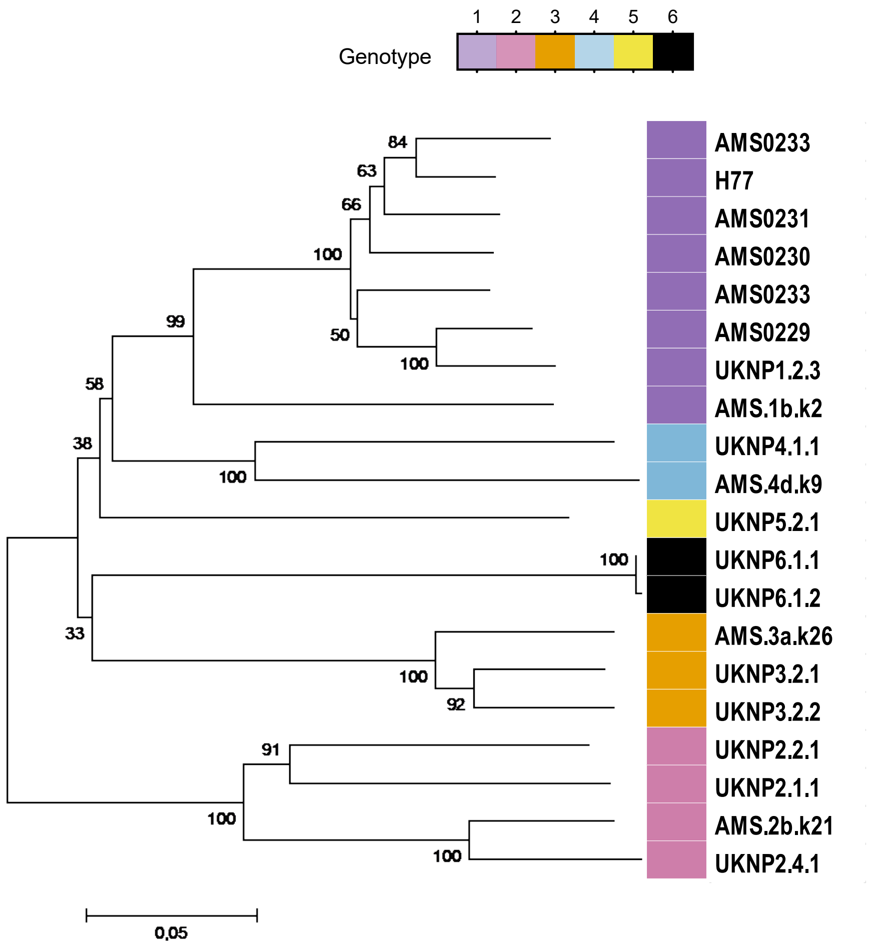

Supplementary Figure 5. Evolutionary relationships of E1E2s from 20 HCVpps inferred using the Neighbor-Joining method. The optimal tree is shown. The percentage of bootstrap test (1000 replicates) are shown next to the nodes. The tree is drawn to scale, with branch lengths in the same units (number of amino acid substitutions per site) as those of the evolutionary distances used to infer the phylogenetic tree. Colored by genotype from 1 to 6. Evolutionary analyses were conducted in MEGA6.

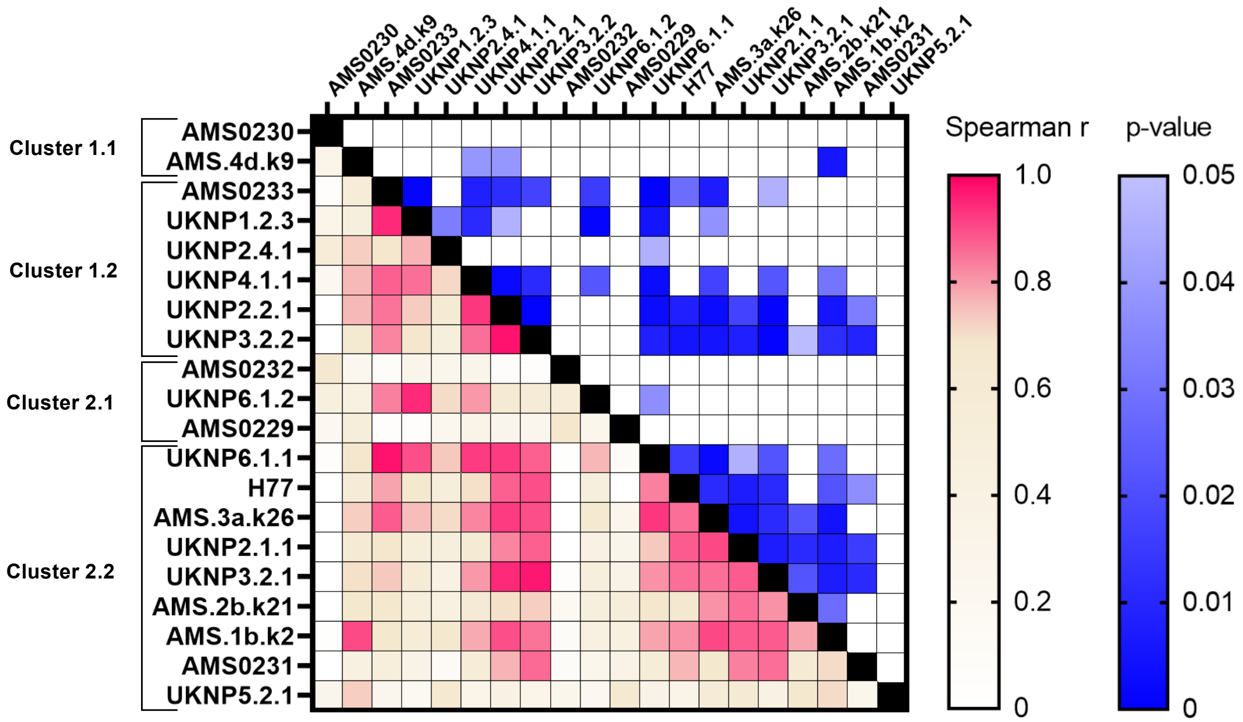

Supplementary Figure 6. Heat map showing pairwise Spearman's correlation matrix for 20 HCVpps based on IC_{50} values of eight mAbs. HCVpps are arranged based on hierarchical clustering of the HCVpps in Figure 1B. Two smaller clusters in cluster 1 and 2 are indicated. Spearman's correlations and p-values are in red and blue, respectively.

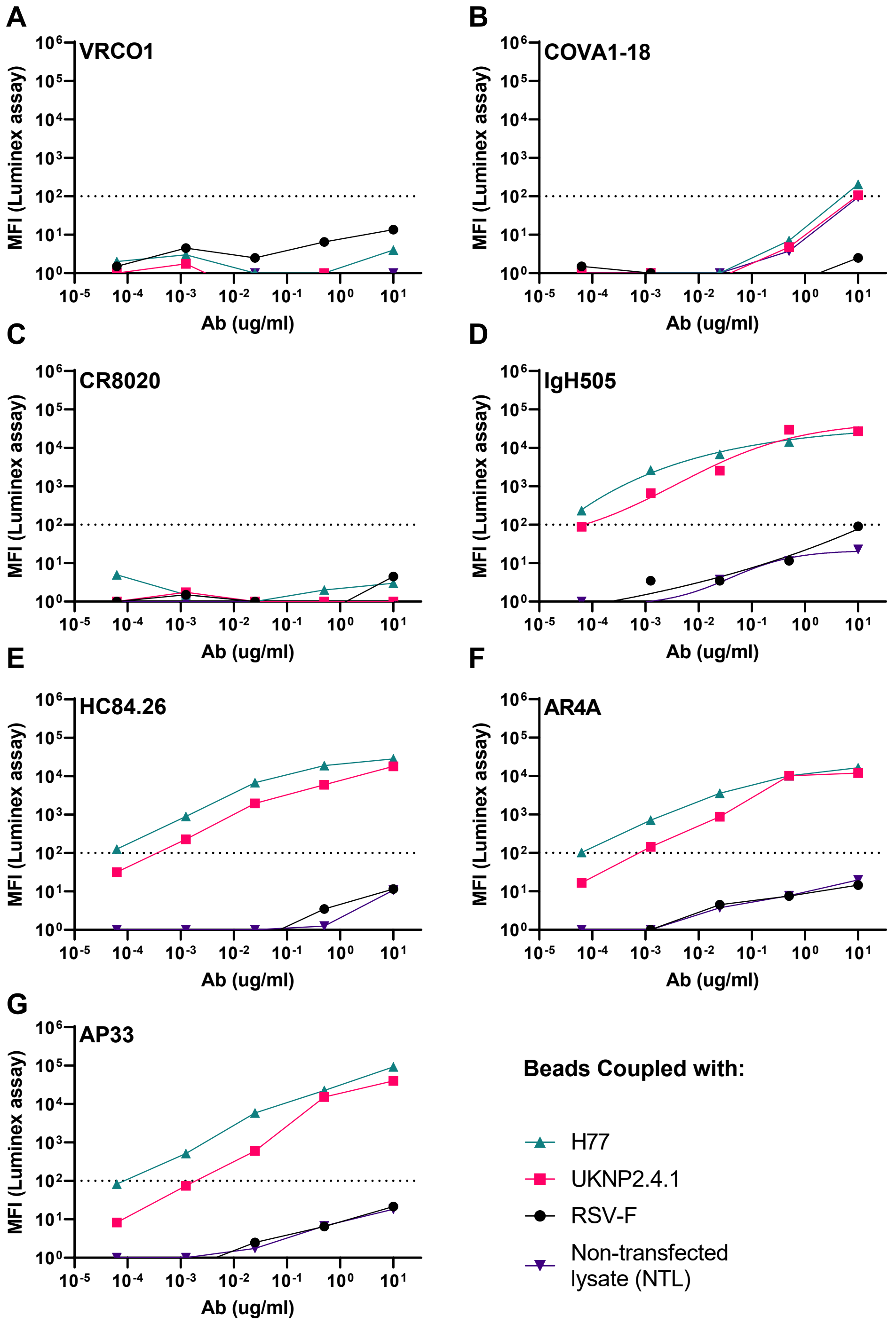

Supplementary Figure 7. Binding results of mAbs to the Luminex beads coupled with mbE1E2s and control proteins. (A) VRC01 (HIV), (B) COVA-18 (Sars-CoV-2) and (C) CR8020 (Influenza), (D) IGH505, (E) HC84.26, (F) AR4A, (G) AP33 mAbs binding to mbE1E2s (H77 and UKNP2.4.1), RSV-F and Non-transfected lysate (NTL) as negative and background beads, respectively. A horizontal dotted lined is shown at 100 MFI.

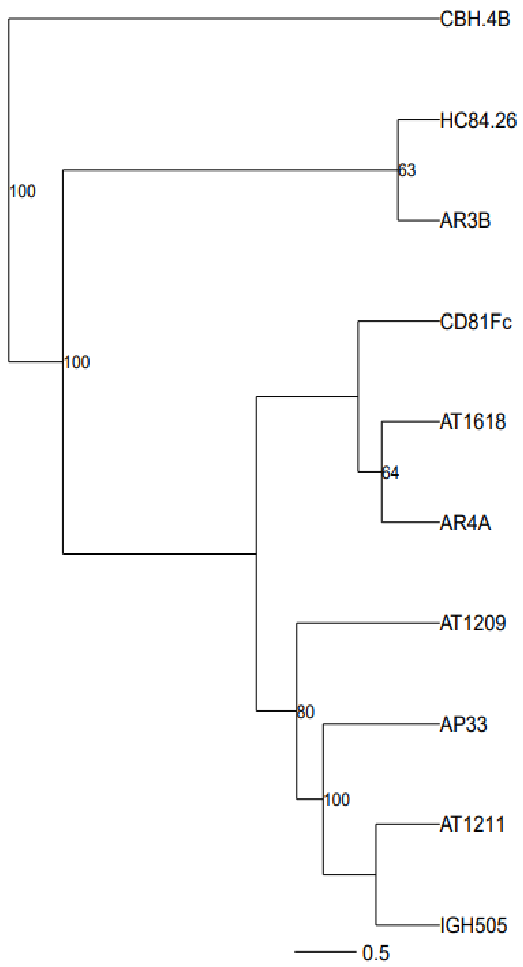

Supplementary Figure 8. Hierarchical clustering of mAbs based on binding (log_{10} MFI) is shown. Bootstrap resampling (1000 iterations) was applied, nodes with support above 50% are shown. Horizontal line represents the scale for the tree branches, which reflects the distance or dissimilarity between data points in median fluorescence intensity (MFI) units.

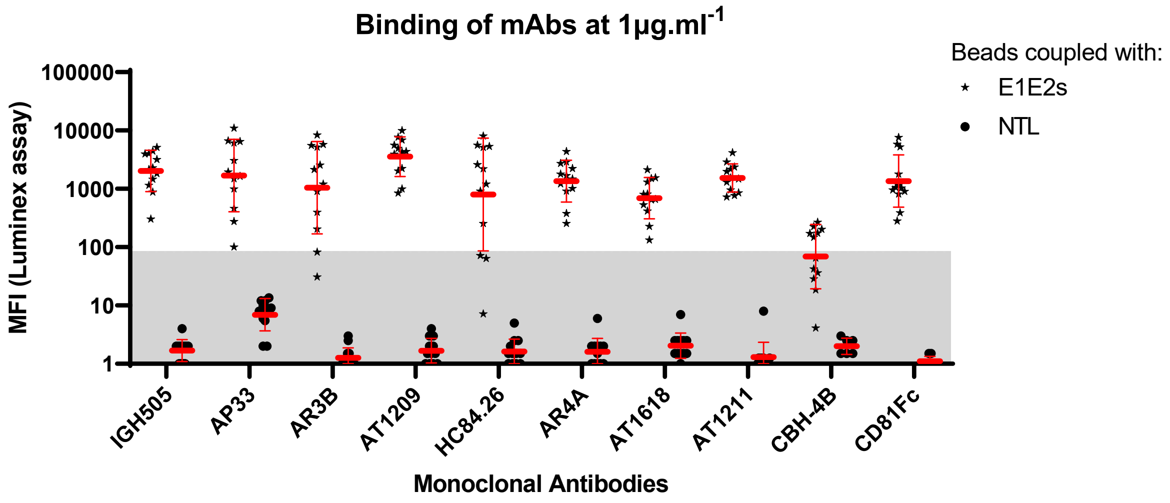

Supplementary Figure 9. Binding (MFI) of mbE1E2s and NTLs per mAb (1µg/ml). GMT and SD of 11 mbE1E2s (stars) and NTL (dots) are shown in red. Gray area is set to 10X the highest GMT for all NTLs (85 MFI) as our binding threshold.

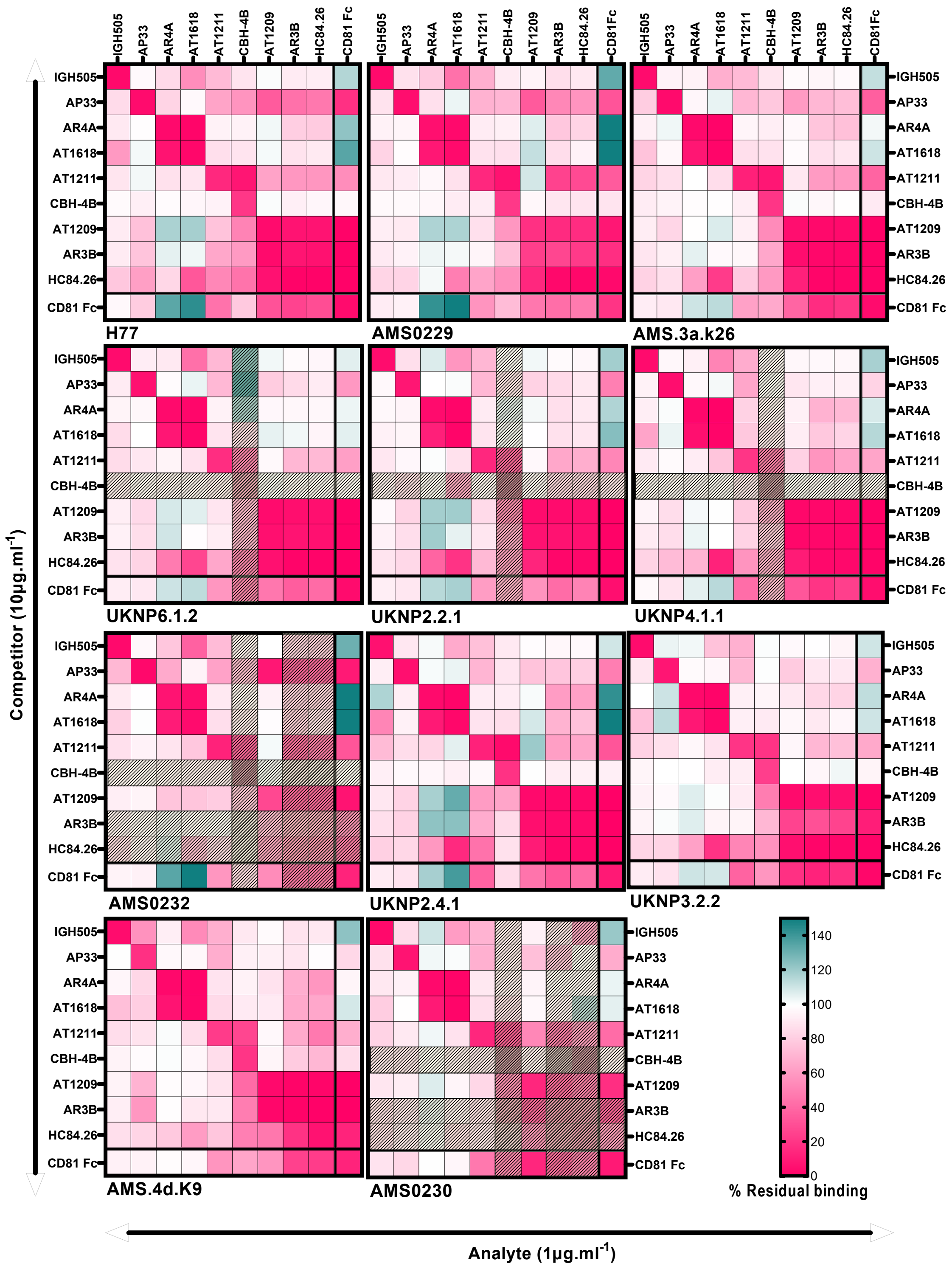

Supplementary Figure 10. Cross competitive residual binding. Luminex matrices for the mean and 11 mbE1E2s are shown. Competitors, unlabeled mAbs (10µg/ml), are on the bottom left and analytes, biotinylated mAbs (1µg/ml), on the top right. Biotinylated mAbs with MFI below our binding threshold are shadowed. Labels indicate the percentage of residual binding relative to the control (no competitor median fluorescence intensity (MFI) equal to 100%) in a color bar from pink to green, where pink indicates strong competition, white no competition and green, binding enhancement. Grey were below binding threshold.

```
                   390        400        410        420        430        440        450        460
           ....|....| ....|....| ....|....| ....|....| ....|....| ....|....| ....|....| ....|....|
H77        VDAETHVTGG SAGRTTAGLV GLLTPGAKQN IQLINTNGSW HINSTALNCN ESLNTGWLAG LFYQHKFNSS GCPERLASCR
AMS0230    .....L.S.. ..A..I..FA S.FRS....D .....S.... ...R...... A..D...V.. .L.H.R.... ..........
AMS0231    .......S.. A.A.NAR... ..FS...Q.. V......... .......... A..D...... ...YNR.D.. ..........

                   470        480        490        500        510        520        530        540
           ....|....| ....|....| ....|....| ....|....| ....|....| ....|....| ....|....| ....|....|
H77        RLTDFAQGWG PISYANGSGL DERPYCWHYP PRPCGIVPAK SVCGPVYCFT PSPVVVGTTD RSGAPTYSWG ANDTDVFVLN
AMS0230    P......... ..G.T...SS .H........ .K........ .......... .......... .......N.. ......L...
AMS0231    P...D..... .........P .Q........ .K........ .......... .......... .......... ..........

                   550        560        570        580        590        600        610        620
           ....|....| ....|....| ....|....| ....|....| ....|....| ....|....| ....|....| ....|....|
H77        NTRPPLGNWF GCTWMNSTGF TKVCGAPPCV IGGVGNNTLL CPTDCFRKHP EATYSRCGSG PWITPRCMVD YPYRLWHYPC
AMS0230    .....Q.... .......S.. .......... .........H .......... .......... .......L.N ..........
AMS0231    .......... .......... .......... .........H .......... .......... .......L.. ..........

                   630        640        650        660        670        680        690        700
           ....|....| ....|....| ....|....| ....|....| ....|....| ....|....| ....|....| ....|....|
H77        TINYTIFKVR MYVGGVEHRL EAACNWTRGE RCDLEDRDRS ELSPLLLSTT QWQVLPCSFT TLPALSTGLI HLHQNIVDVQ
AMS0230    .V........ .......... .......... .......... .......... .......... .......... ..........
AMS0231    .......... .......... .......... ..N....... .......... .......... .......... ..........

                   710        720        730        740
           ....|....| ....|....| ....|....| ....|....| ....|.
H77        YLYGVGSSIA SWAIKWEYVV LLFLLLADAR VCSCLWMMLL ISQAEA
AMS0230    .........V .......... .......... .......... ......
AMS0231    ........V. .......... .......... .......... ......
```

Supplementary Figure 11. Alignment of HCV E2 protein for AMS0230 and AMS0231 to the reference strain H77. Similar amino acids compared to H77 are indicated with a dot.
